# Microsatellite profiling of hosts from parasite-extracted DNA illustrated with raccoons (*Procyon lotor*) and their *Baylisascaris procyonis* roundworms

**DOI:** 10.1186/s13071-023-05703-6

**Published:** 2023-02-25

**Authors:** Alain C. Frantz, Stéphanie Lippert, Mike Heddergott

**Affiliations:** Musée National d’Histoire Naturelle, Luxembourg, Luxembourg

**Keywords:** *Baylisascaris procyonis*, Genetic profiling, Genotyping errors, Microsatellite loci, *Procyon lotor*

## Abstract

**Background:**

Important information on movement pathways and introduction routes of invasive parasites can be obtained by comparing the genetic makeup of an invader with its spatial genetic structure in other distribution areas. Sometimes, the population genetic structure of the host might be more informative than that of the parasite itself, and it is important to collect tissue samples of both host and parasite. However, host tissue samples are frequently not available for analysis. We aimed to test whether it is possible to generate reliable microsatellite profiles of host individuals by amplifying DNA extracted from a nematode parasite, using the raccoon (*Procyon lotor*) and the raccoon roundworm (*Baylisascaris procyonis*) as a test case.

**Methods:**

Between 2020 and 2021, we collected tissue as well as a single roundworm each from 12 raccoons from central Germany. Both the raccoon and the roundworm DNA extracts were genotyped using 17 raccoon-specific microsatellite loci. For each roundworm DNA extract, we performed at least eight amplification reactions per microsatellite locus.

**Results:**

We extracted amplifiable raccoon DNA from all 12 roundworms. We obtained at least two amplification products for 186 of the 204 possible genotypes. Altogether 1077 of the 1106 genotypes (97.4%) matched the host-DNA derived reference genotypes and thus did not contain genotyping errors. Nine of the 12 roundworm-derived genetic profiles matched the reference profiles from the raccoon hosts, with one additional genetic profile containing genotyping errors at a single locus. The remaining two genetic profiles were deemed unsuitable for downstream analysis because of genotyping errors and/or a high proportion of missing data.

**Conclusions:**

We showed that reliable microsatellite-based genetic profiles of host individuals can be obtained by amplifying DNA extracted from a parasitic nematode. Specifically, the approach can be applied to reconstruct invasion pathways of roundworms when samples of the raccoon hosts are lacking. Further research should assess whether this method can be replicated in smaller species of parasitic nematodes and other phyla of parasites more generally.

**Graphical Abstract:**

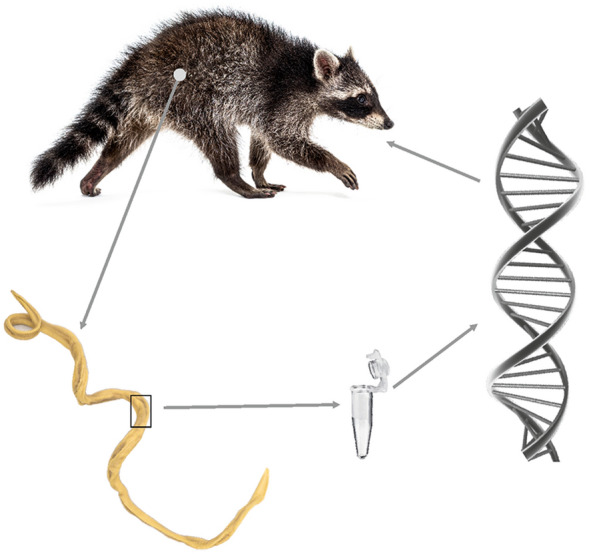

**Supplementary Information:**

The online version contains supplementary material available at 10.1186/s13071-023-05703-6.

## Background

Extensive human-mediated translocation of non-native animals and plants concurrently increases the risk of introducing exotic pathogens into new areas [1, 2]. Parasite invasions therefore represent a growing problem for the natural environment, human and animal health and agriculture [3, 4]. Parasitic nematodes are expected to be frequently translocated alongside their hosts since they are widespread and many host species are infested by more than one species of roundworm [1, 5]. After introduction, non-native nematodes can become highly invasive and serious causative agents of destructive disease [4, 6].

To design effective control and prevention strategies against alien species in general, it is important to gain an understanding of different aspects that contribute to invasion success. One such factor is the identification of invasion routes [7]. These geographic pathways followed by organisms from their source to their introduction site can provide critical information to limit propagule pressure and prevent successful establishment [8]. Important information regarding movement pathways and invasion routes may be gained by comparing the genetic makeup of an invader with the spatial genetic structure of the species in other distribution areas [9–12].

It is often assumed that the population genetic structure of parasites must correspond to that of their hosts because of parasite dispersal being dependent on their more vagile hosts [13–15]. Gene flow patterns often differ between hosts and parasites, however. While the genetic structure of a parasite can be more pronounced than that of the host [15, 16], dispersal rates of parasites are frequently higher than those of their hosts, resulting in significantly lower genetic differentiation in the parasite than the host [14, 15]. When trying to reconstruct geographic introduction pathways of parasitic nematodes, the population genetic structure of the host might therefore be more informative than that of the parasite itself.

When planning to reconstruct geographic introduction pathways of parasitic nematodes, it is thus important to collect tissue samples of both host and parasite. Many historical nematode collections are unlikely to contain tissue samples of the host, however. Except for studies that explicitly compare the genetic structure of parasites and hosts [13, 14], most population genetic studies of parasitic nematodes do not mention that host DNA was sampled (see [15] and references therein). In some cases, it may not be possible to link a parasite to a particular host individual [17]. It has been noted that host DNA can be amplified from adult parasites, for example in studies where parasite microsatellite have been developed [18, 19]. It would therefore be important to be able to generate genetic profiles of hosts based on DNA extracted from parasites.

The raccoon roundworm (*Baylisascaris procyonis*) is a nematode parasite of the gastrointestinal tract of raccoons (*Procyon lotor*) [20]. Via their scats, infested raccoons can expel millions of parasite eggs that, under the right environmental conditions, will remain infectious for years [21]. While usually benign in the raccoon, *B. procyonis* infestations can be fatal in humans and other paratenic hosts [20]. Raccoons and raccoon roundworms are both present in Europe as a result of joint translocations from their native North American range [22]. Raccoons are particularly abundant in Germany [23] where they have increasingly spread into urban areas and come into close contact with humans and domestic animals [24, 25]. Consequently, according to the World Health Organisation baylisascariasis is a zoonosis ‘with current and potentially increasing impact’ in Europe [26]. Raccoons are included in the list of Invasive Alien Species of Union concern [27] and subjected to restrictions and management measures by law [28].

While not all European raccoon populations are parasitised by *B. procyonis*, the roundworm is currently increasing its geographic distribution [29–32]. For effective eradication and/or management actions, it is important to understand whether the parasite was introduced through a distinct founder event or through natural dispersal of infected raccoons [31]. For example, while the first known Austrian roundworm originated from a nearby German population [30], the roundworms in the southern Netherlands originated from a distinct introduction event [31]. However, since the genetic diversity of *B. procyonis* in its introduced range is low, both studies needed to analyse the genetic make-up of the raccoon hosts to infer the origin of the roundworms with certainty [30, 31].

The aim of the present work was to test whether it is possible to generate reliable microsatellite profiles of host individuals by amplifying DNA extracted from a nematode parasite, using the raccoon and the raccoon roundworm as a test case. In addition to a general proof of concept, the information might become relevant in situations where the geographic origin of invading roundworms needs to be determined but where no DNA samples of the raccoon hosts are available.

## Methods

Between 2020 and 2021, we collected large roundworms (> 5 cm) from 12 raccoons that were harvested or road-killed in central Germany (Table 1). We stored the worms and a piece of raccoon muscle tissue in 96% absolute ethanol.

**Table 1:** Geographic origin and characteristics of the raccoon (*Procyon lotor*) hosts from which roundworms (*Baylisascaris procyonis*) were collected for the present analysis

Raccoon id	Sampling date	Geographic origin				Sex	Age	Number of roundworms
		Admin. district	Commune	Longitude (E)	Latitude (N)			
MNHNL120341	25/03/2021	Salzlandkreis	Bernburg (Saale)	11.714519	51.789665	Female	Adult	2
MNHNL120342	08/09/2020	Harz	Thale	11.042805	51.683359	Male	Adult	3
MNHNL120345	17/11/2020	Harz	Halberstadt	10.935173	51.925756	Female	Adult	6
MNHNL120352	21/10/2020	Harz	Harzgerode	11.197901	51.688745	Male	Juvenile	12
MNHNL120368	07/10/2020	Saalekreis	Leuna	12.052925	51.326509	Male	Adult	5
MNHNL120371	21/09/2020	Saalekreis	Leuna	12.023764	51.326536	Male	Adult	5
MNHNL120381	04/09/2020	Harz	Harzgerode	11.128528	51.606437	Female	Juvenile	6
MNHNL120391	30/09/2020	Harz	Quedlinburg	11.171637	51.801078	Female	Adult	5
MNHNL120402	27/04/2020	Stendal	Bismark (Altmark)	11.530495	52.69402	Male	Adult	10
MNHNL120403	13/10/2020	Altmarkkreis Salzwedel	Klötze	11.198502	52.639991	Male	Adult	5
MNHNL120405	19/10/2020	Harz	Halberstadt	10.935173	51.925756	Female	Adult	20
MNHNL120406	06/03/2020	Salzlandkreis	Hecklingen	11.559935	51.844846	Female	Adult	2

Following the manufacturer’s instructions, we used the DNeasy 96 blood and tissue kit (Qiagen, Hilden, Germany) to extract DNA from one roundworm sample per raccoon. We digested an approximately 1-cm-large fragment that included the digestive apparatus of the worm with a final elution volume of 50 μl. We used an ammonium acetate-based salting-out method to extract DNA from the raccoon tissue samples [33]. DNA extracts were quantified with a Drop-Sense 16 spectrophotometer (Trinean, Gentbrugge, Belgium).

Both the raccoon and the roundworm DNA extracts were genotyped using 17 raccoon-specific microsatellite loci [34–36] that were amplified in two multiplex PCRs. Multiplex 1 contained loci PLOT-02, PLOT-05, PLOT-06, PLOT-07, PLOT-08, PLOT-10, PLOT-11, PLOT-13 and PLO3-86. Multiplex 2 contained loci PLM01, PLM03, PLOM2, PLO-M3, PLO-M17, PLO-M20, PLO2-14 and PLO2-117. Each PCR contained 1× GoTaq Master Mix (Promega, Walldorf, Germany) and between 0.1 and 0.4 μM of each primer. PCR conditions for Multiplex 1 were as follows: After a 5-min denaturation at 95 °C, the PCR consisted of 35 cycles of denaturation at 95 °C for 30 s, annealing at 61 °C for 90 s and an extension at 72 °C for 90 s. The PCR was ended with a final extension for 10 min at 68 °C. The same PCR conditions were used for Multiplex 2, except that a touchdown profile was used. The initial annealing temperature of 60 °C was reduced by one degree every cycle for five cycles, followed by 30 cycles of annealing at 55 °C.

While the roundworms were expected to contain raccoon material in their digestive tracts, the concentration of the raccoon-derived DNA was presumed to be low. We therefore added 2 μl of the 50 μl undiluted roundworm DNA extract to each PCR. Negative controls were included at every stage of the process to monitor contamination. Similar to other DNA extracts that are low in quality and quantity, such as faecal DNA, genotyping errors are likely to occur during PCR when using roundworm-extracted DNA to genotype the raccoon host [37, 38]. Genotyping errors include ‘allelic dropout’, where one allele of a heterozygous genotype is not amplified, and ‘false alleles’, where an artificial allele is generated during the PCR as a result of slippage during the initial PCR cycles [39]. For each roundworm DNA extract, we therefore performed eight amplification reactions per microsatellite multiplex. Loci that did not give rise to an amplification product were amplified a further three times in singleplex PCRs (using the same reaction conditions as described above, but with 0.2 μM of the given primer), except in the case of one sample that had missing data at nine loci. Here, the loci with missing data were amplified in two further singleplex PCRs because of the limited quantity of DNA extract. A genotype obtained for a locus was accepted as reliable if an identical result was obtained at least twice and if there was a match with the reference genotype derived from the DNA of the raccoon host. We genotyped the raccoons in duplicate in the three cases where we observed mismatches between the raccoon- and the roundworm-derived profiles (see Additional file 1: Table S1).

## Results

**Fig. 1:**
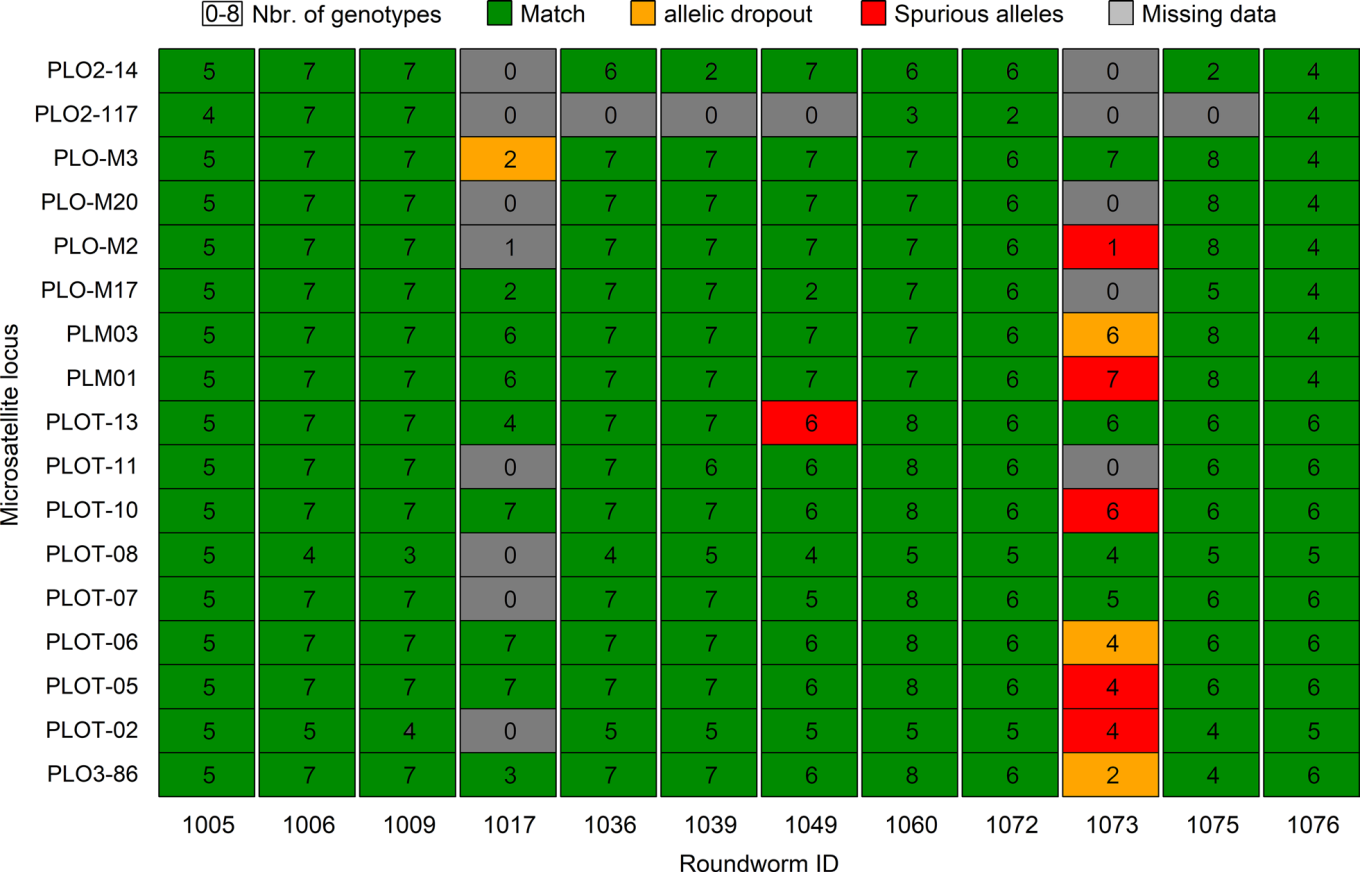
Genotyping success of raccoon (*Procyon lotor*) microsatellite loci when amplified from DNA extracts obtained from roundworm (*Baylisascaris procyonis*) host. We performed repeated genotyping to ensure reliable genetic profiles. Nbr. of genotypes: The number of genotypes generated. Match: The generated genotypes matched the corresponding genotype in a reference profile generated from host DNA. Allelic dropout: Based on comparison with the reference profile, at least one of the genotypes contained an instance of allelic dropout. Spurious alleles: Based on comparison with the reference profile, at least one of the genotypes contained a spurious allele. Missing data: fewer than two genotypes were generated for this locus. All genotypes are given in Additional file 1

We extracted amplifiable raccoon DNA from all 12 roundworms. We generated 1106 raccoon genotypes from these 12 samples, with the total number of genotypes per roundworm sample varying between 45 and 116 (average 92.2; Fig. 1, Additional file 1: Table S1). We generated between zero and eight genotypes per locus per sample, with the average number of genotypes generated per locus varying between 2.6 and 6.9 between samples (Fig. 1, Additional file 1: Table S1). We obtained at least two amplification products for 186 of the 204 possible genotypes (12 samples, 17 loci). Altogether 1077 of the 1106 genotypes (97.4%) matched the reference genotype obtained from the raccoon host and did not contain genotyping errors (Fig. 1, Additional file 1: Table S1). In contrast, based on the reference genotypes, 6 genotypes had a case of allelic drop-out and 22 genotypes contained a spurious allele.

Nine of the 12 genetic profiles did not contain any genotyping errors (Fig. 1, Additional file 1: Table S1). In the case of six roundworm DNA extracts, we managed to obtain a complete consensus genotype that exactly matched the corresponding host reference profile. A further three samples exactly matched their host reference profile, but had missing data at one locus. One further sample had spurious alleles at one locus (leading to an erroneous consensus genotype; Additional file 1) and missing data at another one. The remaining two genetic profiles were problematic. While one of these genetic profiles had missing genotype data at eight loci and genotyping errors at one further locus, the other profile had missing genotype data at six loci and genotyping errors at a further seven loci. In the case of the latter profile, repeated occurrence of the same spurious alleles would have led to five erroneous genotypes in the final consensus profile (which did not match another reference profile; Additional file 1).

## Discussion

We aimed to test whether reliable microsatellite-based genetic profiles of host individuals can be obtained by amplifying DNA extracted from nematode parasites and tested the approach using the raccoon and its roundworm parasite. In most cases, we obtained DNA of sufficient quality and quantity to allow efficient and error-free genotyping/genetic profiling of the host from parasite-derived DNA. Most genotypes (97.4%) matched the host-derived reference genotype and thus did not contain any apparent genotyping errors. DNA samples obtained from non-invasively collected samples, such as faeces, for instance, generally have higher genotyping error rates [39]. It is thus likely that we obtained a higher target-DNA yield or that the target DNA was less degraded than is the case with many faecal DNA extracts. Due to the high success rate of the genotyping, nine of the 12 roundworm-derived DNA samples gave rise to error-free genetic profiles of the raccoon hosts, with one additional genetic profile containing genotyping errors at a single locus. However, the remaining two samples would not be suitable for downstream analysis because of genotyping errors and/or a high proportion of missing data.

While our method provided promising results, the genetic profiles derived from two samples proved to be problematic. Because all DNA extracts were derived from large worms, these two samples did not a priori contain less host DNA. In the case of one of these samples, spurious alleles would have led to five erroneous genotypes in the final consensus profile. It is unusual to generate the same spurious alleles repeatedly [40]. We genotyped the loci in the reference profile in duplicate to ensure that the discrepancy between the host-derived reference profile and the consensus profile generated from parasite DNA was not due to genotyping errors in the reference profile. Since the roundworms develop from larvae to adults in the raccoon host, it is unlikely that the roundworm contained DNA of a raccoon other than its host. In addition, because the profile did not match another reference profile, we can exclude sample mix-up. The second profile with a large proportion of missing data only had genotyping errors at one of the loci for which genotypes obtained. Nevertheless, to avoid erroneous genetic profiles, it would probably be safer to exclude profiles from downstream analysis that have missing data at a larger number of loci and that thus originated from extracts with reduced DNA quantity or quality.

Our method was successful in generating genetic profiles of host individuals using DNA extracted from their parasites. However, we tested the approach with large nematodes that presumably contained relatively large amounts of ingested host tissue. Further research will show whether the approach is similarly successful with other smaller species of parasitic nematode and other phyla of parasites more generally. Genotyping of hosts from hematophagous arthropods is likely to remain challenging because of the low quality and quantity of nuclear DNA extracted from blood meals as well as the presence of DNA mixtures derived from multiple hosts [41]. Our samples were collected and stored rapidly after the death of the host. It remains to be seen whether genotyping of hosts from parasite-derived DNA will prove similarly successful with museum specimens and other older samples.

The population genetic structure of the raccoon in western and central Europe is more pronounced than that of its roundworm parasite [19, 31]. Studies attempting to reconstruct the geographic invasion pathways of the raccoon roundworm thus also had to analyse the genetic makeup of the raccoon hosts to infer the origin of the parasites with certainty [30, 31]. A potential flaw with this approach is that it assumes that the first animal shown to be infested with roundworms had also carried the parasite into the region/country of interests, while it may have become infested after contact with other animals or through environmental contamination. However, this caveat also applies to the situation where tissue samples are available from both host and parasites. Moreover, given that parasite-extracted DNA will contain a mixture of host and parasite DNA, the method can only be used to amplify host-specific markers.

## Conclusions

We showed that, in principle, reliable microsatellite-based genetic profiles of host individuals can be obtained by amplifying DNA extracted from nematode parasites. Specifically, this approach could be applied to reconstruct introduction pathways of roundworms when samples of the raccoon hosts are lacking. A certain caution is required to avoid erroneous genetic profiles and researchers may want to exclude genetic profiles with a larger proportion of missing data from downstream analysis. We therefore recommend following methodologies developed for other low quality/quantity DNA samples, e.g. faecal samples, whereby multiple rounds of amplifications reactions are used to obtain a consensus genotypes. Further research should assess whether this method can be replicated in smaller species of parasitic nematodes and, more generally, in other phyla of parasites.

## Supplementary Information

**Additional file 1**: **Table S1**: Results of the 1106 DNA amplification reactions performed to generate genetic profiles of 12 raccoons (*Procyon lotor*) based on DNA extracted from their roundworm (*Baylisascaris procyonis*) parasites. The genotypes in bold correspond to reference genotypes generated from DNA extracted from host tissue. The host-derived DNA was genotyped in duplicate in the three cases where we observed mismatches between the raccoon- and roundworm-derived profiles. Parasite-derived genotypes that are in italics and underlined contain a genotyping error (allelic dropout or false allele). All loci were amplified at least eight times. Failed reactions have not been included.

## Data Availability

All data generated or analysed during this study are included in this published article and its Additional information files.

## Abbreviation

PCR: Polymerase chain reaction
