# Supplementary material for: Microsatellite profiling of hosts from parasite-extracted DNA illustrated with raccoons (Procyon lotor) and their Baylisascaris procyonis roundworms
Source: Parasit Vectors. 2023 Feb 25;16:76. doi: 10.1186/s13071-023-05703-6 (PMC9960475; doi:10.1186/s13071-023-05703-6)
Supplement: Supplementary file 1 — Additional file 1: Table S1: Results of the 1106 DNA amplification reactions performed to generate genetic profiles of 12 raccoons (Procyon lotor) based on DNA extracted from their roundworm (Baylisascaris procyonis) parasites. The genotypes in bold correspond to reference genotypes generated from DNA extracted from host tissue. The host-derived DNA was genotyped in duplicate in the three cases where we observed mismatches between the raccoon- and roundworm-derived profiles. Parasite-derived genotypes that are in italics and underlined contain a genotyping error (allelic dropout or false allele). All loci were amplified at least eight times. Failed reactions have not been included. [file 13071_2023_5703_MOESM1_ESM.docx]

Additional file 1: Text S1: Results of the 1106 DNA amplification reactions performed to generate genetic profiles of 12 raccoons (*Procyon lotor*) based on DNA extracted from their roundworm (*Baylisascaris procyonis*) parasites. The genotypes in bold correspond to reference genotypes generated from DNA extracted from host tissue. The host-derived DNA was genotyped in duplicate in the three cases where we observed mismatches between the raccoon- and the roundworm-derived profiles. Parasite-derived genotypes that are in italic and underlined contain a genotyping error (allelic dropout or false allele). All loci were amplified at least eight times. Failed reactions have not been included

| Indiviual ID | PLO3-86 | PLOT-02 | PLOT-05 | PLOT-06 | PLOT-07 | PLOT-08 | PLOT-10 | PLOT-11 | PLOT-13 | PLM01 | PLM03 | PLO-M17 | PLO-M2 | PLO-M20 | PLO-M3 | PLO2-117 | PLO2-14 |
| --- | --- | --- | --- | --- | --- | --- | --- | --- | --- | --- | --- | --- | --- | --- | --- | --- | --- |
| **P.l.M1005** | **339 339** | **201 201** | **128 129** | **161 165** | **209 209** | **247 251** | **146 158** | **198 200** | **343 343** | **93 103** | **126 126** | **224 232** | **281 285** | **189 209** | **266 278** | **312 312** | **244 244** |
|  | 339 339 | 201 201 | 128 129 | 161 165 | 209 209 | 247 251 | 146 158 | 198 200 | 343 343 | 93 103 | 126 126 | 224 232 | 281 285 | 189 209 | 266 278 | 312 312 | 244 244 |
|  | 339 339 | 201 201 | 128 129 | 161 165 | 209 209 | 247 251 | 146 158 | 198 200 | 343 343 | 93 103 | 126 126 | 224 232 | 281 285 | 189 209 | 266 278 | 312 312 | 244 244 |
|  | 339 339 | 201 201 | 128 129 | 161 165 | 209 209 | 247 251 | 146 158 | 198 200 | 343 343 | 93 103 | 126 126 | 224 232 | 281 285 | 189 209 | 266 278 | 312 312 | 244 244 |
|  | 339 339 | 201 201 | 128 129 | 161 165 | 209 209 | 247 251 | 146 158 | 198 200 | 343 343 | 93 103 | 126 126 | 224 232 | 281 285 | 189 209 | 266 278 | 312 312 | 244 244 |
|  | 339 339 | 201 201 | 128 129 | 161 165 | 209 209 | 247 251 | 146 158 | 198 200 | 343 343 | 93 103 | 126 126 | 224 232 | 281 285 | 189 209 | 266 278 |  | 244 244 |
| **P.l.M1006** | **339 339** | **185 201** | **125 128** | **161 165** | **217 217** | **243 243** | **158 158** | **198 200** | **343 343** | **89 93** | **126 135** | **212 224** | **281 313** | **193 205** | **270 278** | **318 330** | **240 240** |
|  | 339 339 | 185 201 | 125 128 | 161 165 | 217 217 | 243 243 | 158 158 | 198 200 | 343 343 | 89 93 | 126 135 | 212 224 | 281 313 | 193 205 | 270 278 | 318 330 | 240 240 |
|  | 339 339 | 185 201 | 125 128 | 161 165 | 217 217 | 243 243 | 158 158 | 198 200 | 343 343 | 89 93 | 126 135 | 212 224 | 281 313 | 193 205 | 270 278 | 318 330 | 240 240 |
|  | 339 339 | 185 201 | 125 128 | 161 165 | 217 217 | 243 243 | 158 158 | 198 200 | 343 343 | 89 93 | 126 135 | 212 224 | 281 313 | 193 205 | 270 278 | 318 330 | 240 240 |
|  | 339 339 | 185 201 | 125 128 | 161 165 | 217 217 | 243 243 | 158 158 | 198 200 | 343 343 | 89 93 | 126 135 | 212 224 | 281 313 | 193 205 | 270 278 | 318 330 | 240 240 |
|  | 339 339 | 185 201 | 125 128 | 161 165 | 217 217 |  | 158 158 | 198 200 | 343 343 | 89 93 | 126 135 | 212 224 | 281 313 | 193 205 | 270 278 | 318 330 | 240 240 |
|  | 339 339 |  | 125 128 | 161 165 | 217 217 |  | 158 158 | 198 200 | 343 343 | 89 93 | 126 135 | 212 224 | 281 313 | 193 205 | 270 278 | 318 330 | 240 240 |
|  | 339 339 |  | 125 128 | 161 165 | 217 217 |  | 158 158 | 198 200 | 343 343 | 89 93 | 126 135 | 212 224 | 281 313 | 193 205 | 270 278 | 318 330 | 240 240 |
| **P.l.M1009** | **339 419** | **185 185** | **120 128** | **161 173** | **209 215** | **243 243** | **162 162** | **200 204** | **341 343** | **89 101** | **135 135** | **212 228** | **313 317** | **193 205** | **278 278** | **312 312** | **244 252** |
|  | 339 419 | 185 185 | 120 128 | 161 173 | 209 215 | 243 243 | 162 162 | 200 204 | 341 343 | 89 101 | 135 135 | 212 228 | 313 317 | 193 205 | 278 278 | 312 312 | 244 252 |
|  | 339 419 | 185 185 | 120 128 | 161 173 | 209 215 | 243 243 | 162 162 | 200 204 | 341 343 | 89 101 | 135 135 | 212 228 | 313 317 | 193 205 | 278 278 | 312 312 | 244 252 |
|  | 339 419 | 185 185 | 120 128 | 161 173 | 209 215 | 243 243 | 162 162 | 200 204 | 341 343 | 89 101 | 135 135 | 212 228 | 313 317 | 193 205 | 278 278 | 312 312 | 244 252 |
|  | 339 419 | 185 185 | 120 128 | 161 173 | 209 215 |  | 162 162 | 200 204 | 341 343 | 89 101 | 135 135 | 212 228 | 313 317 | 193 205 | 278 278 | 312 312 | 244 252 |
|  | 339 419 |  | 120 128 | 161 173 | 209 215 |  | 162 162 | 200 204 | 341 343 | 89 101 | 135 135 | 212 228 | 313 317 | 193 205 | 278 278 | 312 312 | 244 252 |
|  | 339 419 |  | 120 128 | 161 173 | 209 215 |  | 162 162 | 200 204 | 341 343 | 89 101 | 135 135 | 212 228 | 313 317 | 193 205 | 278 278 | 312 312 | 244 252 |
|  | 339 419 |  | 120 128 | 161 173 | 209 215 |  | 162 162 | 200 204 | 341 343 | 89 101 | 135 135 | 212 228 | 313 317 | 193 205 | 278 278 | 312 312 | 244 252 |
| **P.l.M1060** | **339 419** | **185 191** | **108 114** | **165 165** | **217 217** | **243 251** | **150 150** | **198 204** | **343 343** | **93 93** | **126 135** | **212 232** | **218 313** | **196 205** | **266 278** | **312 342** | **232 240** |
|  | 339 419 | 185 191 | 108 114 | 165 165 | 217 217 | 243 251 | 150 150 | 198 204 | 343 343 | 93 93 | 126 135 | 212 232 | 218 313 | 196 205 | 266 278 | 312 342 | 232 240 |
|  | 339 419 | 185 191 | 108 114 | 165 165 | 217 217 | 243 251 | 150 150 | 198 204 | 343 343 | 93 93 | 126 135 | 212 232 | 218 313 | 196 205 | 266 278 | 312 342 | 232 240 |
|  | 339 419 | 185 191 | 108 114 | 165 165 | 217 217 | 243 251 | 150 150 | 198 204 | 343 343 | 93 93 | 126 135 | 212 232 | 218 313 | 196 205 | 266 278 | 312 342 | 232 240 |
|  | 339 419 | 185 191 | 108 114 | 165 165 | 217 217 | 243 251 | 150 150 | 198 204 | 343 343 | 93 93 | 126 135 | 212 232 | 218 313 | 196 205 | 266 278 |  | 232 240 |
|  | 339 419 | 185 191 | 108 114 | 165 165 | 217 217 | 243 251 | 150 150 | 198 204 | 343 343 | 93 93 | 126 135 | 212 232 | 218 313 | 196 205 | 266 278 |  | 232 240 |
|  | 339 419 |  | 108 114 | 165 165 | 217 217 |  | 150 150 | 198 204 | 343 343 | 93 93 | 126 135 | 212 232 | 218 313 | 196 205 | 266 278 |  |  |
|  | 339 419 |  | 108 114 | 165 165 | 217 217 |  | 150 150 | 198 204 | 343 343 | 93 93 | 126 135 | 212 232 | 218 313 | 196 205 | 266 278 |  |  |
|  | 339 419 |  | 108 114 | 165 165 | 217 217 |  | 150 150 | 198 204 | 343 343 |  |  |  |  |  |  |  |  |
| **P.l.M1072** | **381 399** | **193 193** | **114 125** | **161 165** | **215 215** | **243 243** | **150 162** | **200 204** | **343 343** | **93 93** | **134 135** | **216 232** | **281 301** | **205 209** | **278 278** | **326 340** | **232 259** |
|  | 381 399 | 193 193 | 114 125 | 161 165 | 215 215 | 243 243 | 150 162 | 200 204 | 343 343 | 93 93 | 134 135 | 216 232 | 281 301 | 205 209 | 278 278 | 326 340 | 232 259 |
|  | 381 399 | 193 193 | 114 125 | 161 165 | 215 215 | 243 243 | 150 162 | 200 204 | 343 343 | 93 93 | 134 135 | 216 232 | 281 301 | 205 209 | 278 278 | 326 340 | 232 259 |
|  | 381 399 | 193 193 | 114 125 | 161 165 | 215 215 | 243 243 | 150 162 | 200 204 | 343 343 | 93 93 | 134 135 | 216 232 | 281 301 | 205 209 | 278 278 |  | 232 259 |
|  | 381 399 | 193 193 | 114 125 | 161 165 | 215 215 | 243 243 | 150 162 | 200 204 | 343 343 | 93 93 | 134 135 | 216 232 | 281 301 | 205 209 | 278 278 |  | 232 259 |
|  | 381 399 | 193 193 | 114 125 | 161 165 | 215 215 | 243 243 | 150 162 | 200 204 | 343 343 | 93 93 | 134 135 | 216 232 | 281 301 | 205 209 | 278 278 |  | 232 259 |
|  | 381 399 |  | 114 125 | 161 165 | 215 215 |  | 150 162 | 200 204 | 343 343 | 93 93 | 134 135 | 216 232 | 281 301 | 205 209 | 278 278 |  | 232 259 |
| **P.l.M1076** | **415 419** | **185 185** | **114 128** | **165 165** | **217 219** | **243 243** | **150 170** | **200 202** | **343 343** | **93 93** | **126 126** | **212 212** | **281 293** | **193 205** | **278 278** | **312 330** | **323 244** |
|  | 415 419 | 185 185 | 114 128 | 165 165 | 217 219 | 243 243 | 150 170 | 200 202 | 343 343 | 93 93 | 126 126 | 212 212 | 281 293 | 193 205 | 278 278 | 312 330 | 232 244 |
|  | 415 419 | 185 185 | 114 128 | 165 165 | 217 219 | 243 243 | 150 170 | 200 202 | 343 343 | 93 93 | 126 126 | 212 212 | 281 293 | 193 205 | 278 278 | 312 330 | 232 244 |
|  | 415 419 | 185 185 | 114 128 | 165 165 | 217 219 | 243 243 | 150 170 | 200 202 | 343 343 | 93 93 | 126 126 | 212 212 | 281 293 | 193 205 | 278 278 | 312 330 | 232 244 |
|  | 415 419 | 185 185 | 114 128 | 165 165 | 217 219 | 243 243 | 150 170 | 200 202 | 343 343 | 93 93 | 126 126 | 212 212 | 281 293 | 193 205 | 278 278 | 312 330 | 232 244 |
|  | 415 419 | 185 185 | 114 128 | 165 165 | 217 219 | 243 243 | 150 170 | 200 202 | 343 343 |  |  |  |  |  |  |  |  |
|  | 415 419 |  | 114 128 | 165 165 | 217 219 |  | 150 170 | 200 202 | 343 343 |  |  |  |  |  |  |  |  |
| **P.l.M1036** | **387 387** | **201 201** | **114 128** | **161 165** | **209 209** | **243 251** | **158 162** | **200 200** | **341 343** | **89 93** | **126 128** | **212 220** | **293 293** | **193 196** | **278 278** | **312 330** | **240 244** |
|  | 387 387 | 201 201 | 114 128 | 161 165 | 209 209 | 243 251 | 158 162 | 200 200 | 341 343 | 89 93 | 126 128 | 212 220 | 293 293 | 193 196 | 278 278 |  | 240 244 |
|  | 387 387 | 201 201 | 114 128 | 161 165 | 209 209 | 243 251 | 158 162 | 200 200 | 341 343 | 89 93 | 126 128 | 212 220 | 293 293 | 193 196 | 278 278 |  | 240 244 |
|  | 387 387 | 201 201 | 114 128 | 161 165 | 209 209 | 243 251 | 158 162 | 200 200 | 341 343 | 89 93 | 126 128 | 212 220 | 293 293 | 193 196 | 278 278 |  | 240 244 |
|  | 387 387 | 201 201 | 114 128 | 161 165 | 209 209 | 243 251 | 158 162 | 200 200 | 341 343 | 89 93 | 126 128 | 212 220 | 293 293 | 193 196 | 278 278 |  | 240 244 |
|  | 387 387 | 201 201 | 114 128 | 161 165 | 209 209 |  | 158 162 | 200 200 | 341 343 | 89 93 | 126 128 | 212 220 | 293 293 | 193 196 | 278 278 |  | 240 244 |
|  | 387 387 |  | 114 128 | 161 165 | 209 209 |  | 158 162 | 200 200 | 341 343 | 89 93 | 126 128 | 212 220 | 293 293 | 193 196 | 278 278 |  | 240 244 |
|  | 387 387 |  | 114 128 | 161 165 | 209 209 |  | 158 162 | 200 200 | 341 343 | 89 93 | 126 128 | 212 220 | 293 293 | 193 196 | 278 278 |  |  |
| **P.l.M1039** | **339 419** | **201 201** | **128 129** | **161 165** | **217 217** | **243 247** | **150 162** | **200 202** | **341 343** | **93 93** | **126 128** | **216 220** | **281 285** | **196 213** | **270 278** | **330 330** | **240 256** |
|  | 339 419 | 201 201 | 128 129 | 161 165 | 217 217 | 243 247 | 150 162 | 200 202 | 341 343 | 93 93 | 126 128 | 216 220 | 281 285 | 196 213 | 270 278 |  | 240 256 |
|  | 339 419 | 201 201 | 128 129 | 161 165 | 217 217 | 243 247 | 150 162 | 200 202 | 341 343 | 93 93 | 126 128 | 216 220 | 281 285 | 196 213 | 270 278 |  | 240 256 |
|  | 339 419 | 201 201 | 128 129 | 161 165 | 217 217 | 243 247 | 150 162 | 200 202 | 341 343 | 93 93 | 126 128 | 216 220 | 281 285 | 196 213 | 270 278 |  |  |
|  | 339 419 | 201 201 | 128 129 | 161 165 | 217 217 | 243 247 | 150 162 | 200 202 | 341 343 | 93 93 | 126 128 | 216 220 | 281 285 | 196 213 | 270 278 |  |  |
|  | 339 419 | 201 201 | 128 129 | 161 165 | 217 217 | 243 247 | 150 162 | 200 202 | 341 343 | 93 93 | 126 128 | 216 220 | 281 285 | 196 213 | 270 278 |  |  |
|  | 339 419 |  | 128 129 | 161 165 | 217 217 |  | 150 162 | 200 202 | 341 343 | 93 93 | 126 128 | 216 220 | 281 285 | 196 213 | 270 278 |  |  |
|  | 339 419 |  | 128 129 | 161 165 | 217 217 |  | 150 162 |  | 341 343 | 93 93 | 126 128 | 216 220 | 281 285 | 196 213 | 270 278 |  |  |
| **P.l.M1075** | **391 419** | **185 201** | **108 125** | **165 165** | **209 215** | **247 251** | **162 162** | **200 200** | **343 343** | **89 93** | **126 135** | **212 216** | **285 293** | **189 189** | **278 278** | **318 336** | **232 240** |
|  | 391 419 | 185 201 | 108 125 | 165 165 | 209 215 | 247 251 | 162 162 | 200 200 | 343 343 | 89 93 | 126 135 | 212 216 | 285 293 | 189 189 | 278 278 |  | 232 240 |
|  | 391 419 | 185 201 | 108 125 | 165 165 | 209 215 | 247 251 | 162 162 | 200 200 | 343 343 | 89 93 | 126 135 | 212 216 | 285 293 | 189 189 | 278 278 |  | 232 240 |
|  | 391 419 | 185 201 | 108 125 | 165 165 | 209 215 | 247 251 | 162 162 | 200 200 | 343 343 | 89 93 | 126 135 | 212 216 | 285 293 | 189 189 | 278 278 |  |  |
|  | 391 419 | 185 201 | 108 125 | 165 165 | 209 215 | 247 251 | 162 162 | 200 200 | 343 343 | 89 93 | 126 135 | 212 216 | 285 293 | 189 189 | 278 278 |  |  |
|  |  |  | 108 125 | 165 165 | 209 215 | 247 251 | 162 162 | 200 200 | 343 343 | 89 93 | 126 135 | 212 216 | 285 293 | 189 189 | 278 278 |  |  |
|  |  |  | 108 125 | 165 165 | 209 215 |  | 162 162 | 200 200 | 343 343 | 89 93 | 126 135 |  | 285 293 | 189 189 | 278 278 |  |  |
|  |  |  |  |  |  |  |  |  |  | 89 93 | 126 135 |  | 285 293 | 189 189 | 278 278 |  |  |
|  |  |  |  |  |  |  |  |  |  | 89 93 | 126 135 |  | 285 293 | 189 189 | 278 278 |  |  |
|  |  |  |  |  |  |  |  |  |  |  |  |  |  |  |  |  |  |
|  |  |  |  |  |  |  |  |  |  |  |  |  |  |  |  |  |  |
|  |  |  |  |  |  |  |  |  |  |  |  |  |  |  |  |  |  |
|  |  |  |  |  |  |  |  |  |  |  |  |  |  |  |  |  |  |
|  |  |  |  |  |  |  |  |  |  |  |  |  |  |  |  |  |  |
| **P.l.M1049** | **399 415** | **185 197** | **125 128** | **165 165** | **215 215** | **243 251** | **150 162** | **198 204** | **343 343** | **89 93** | **134 135** | **232 232** | **281 297** | **189 205** | **266 278** | **312 316** | **240 240** |
|  | **399 415** | **185 197** | **125 128** | **165 165** | **215 215** | **243 251** | **150 162** |  | **343 343** | **89 83** | **134 135** |  |  |  | **266 278** |  |  |
|  | 399 415 | 185 197 | 125 128 | 165 165 | 215 215 | 243 251 | 150 162 | 198 204 | 341 343 | 89 93 | 134 135 | 232 232 | 281 297 | 189 205 | 266 278 |  | 240 240 |
|  | 399 415 | 185 197 | 125 128 | 165 165 | 215 215 | 243 251 | 150 162 | 198 204 | *343 343* | 89 93 | 134 135 | 232 232 | 281 297 | 189 205 | 266 278 |  | 240 240 |
|  | 399 415 | 185 197 | 125 128 | 165 165 | 215 215 | 243 251 | 150 162 | 198 204 | 341 343 | 89 93 | 134 135 |  | 281 297 | 189 205 | 266 278 |  | 240 240 |
|  | 399 415 | 185 197 | 125 128 | 165 165 | 215 215 | 243 251 | 150 162 | 198 204 | *343 343* | 89 93 | 134 135 |  | 281 297 | 189 205 | 266 278 |  | 240 240 |
|  | 399 415 | 185 197 | 125 128 | 165 165 | 215 215 |  | 150 162 | 198 204 | 343 343 | 89 93 | 134 135 |  | 281 297 | 189 205 | 266 278 |  | 240 240 |
|  | 399 415 |  | 125 128 | 165 165 |  |  | 150 162 | 198 204 | 343 343 | 89 93 | 134 135 |  | 281 297 | 189 205 | 266 278 |  | 240 240 |
|  |  |  |  |  |  |  |  |  |  | 89 93 | 134 135 |  | 281 297 | 189 205 | 266 278 |  | 240 240 |
| **P.l.M1017** | **419 419** | **201 201** | **108 114** | **165 165** | **209 215** | **239 243** | **158 158** | **200 202** | **343 343** | **93 103** | **126 134** | **212 212** | **281 313** | **189 205** | **270 278** | **314 340** | **240 244** |
|  | **419 419** |  | **108 114** | **165 165** | **209 215** | **238 243** | **158 158** |  | **343 343** | **93 103** | **126 134** | **212 212** | **281 313** | **189 205** | **270 278** |  | **240 244** |
|  | 419 419 |  | 108 114 | 165 165 |  |  | 158 158 |  | 343 343 | 93 103 | 126 134 | 212 212 | 281 313 |  | *270 270* |  |  |
|  | 419 419 |  | 108 114 | 165 165 |  |  | 158 158 |  | 343 343 | 93 103 | 126 134 | 212 212 |  |  | *278 278* |  |  |
|  | 419 419 |  | 108 114 | 165 165 |  |  | 158 158 |  | 343 343 | 93 103 | 126 134 |  |  |  |  |  |  |
|  |  |  | 108 114 | 165 165 |  |  | 158 158 |  | 343 343 | 93 103 | 126 134 |  |  |  |  |  |  |
|  |  |  | 108 114 | 165 165 |  |  | 158 158 |  |  | 93 103 | 126 134 |  |  |  |  |  |  |
|  |  |  | 108 114 | 165 165 |  |  | 158 158 |  |  | 93 103 | 126 134 |  |  |  |  |  |  |
|  |  |  | 108 114 | 165 165 |  |  | 158 158 |  |  |  |  |  |  |  |  |  |  |
| **P.l.M1073** | **339 419** | **187 201** | **128 128** | **165 173** | **215 215** | **243 243** | **162 162** | **198 200** | **343 343** | **89 89** | **126 135** | **212 232** | **297 301** | **193 205** | **278 278** | **326 330** | **244 256** |
|  | **339 419** | **187 201** | **128 128** | **165 173** | **215 215** | **243 243** | **162 162** |  | **343 343** | **89 89** | **126 135** |  | **297 301** | **193 205** | **278 278** |  |  |
|  | *339 339* | *193 193* | *114 128* | 165 173 | 215 215 | 243 243 | 162 162 |  | 343 343 | *89 93* | *135 135* |  | *281 301* |  | 278 278 |  |  |
|  | *339 339* | *193 193* | *114 128* | *165 165* | 215 215 | 243 243 | *150 162* |  | 343 343 | *89 93* | 126 135 |  |  |  | 278 278 |  |  |
|  |  | *193 193* | *114 128* | 165 173 | 215 215 | 243 243 | *150 162* |  | 343 343 | *89 93* | 126 135 |  |  |  | 278 278 |  |  |
|  |  | *193 193* | *114 125* | *161 165* | 215 215 | 243 243 | 162 162 |  | 343 343 | *89 93* | 126 135 |  |  |  | 278 278 |  |  |
|  |  |  |  |  | 215 215 |  | *150 162* |  | 343 343 | *89 93* | 126 135 |  |  |  | 278 278 |  |  |
|  |  |  |  |  |  |  | *150 162* |  | 343 343 | *89 93* | 126 135 |  |  |  | 278 278 |  |  |
|  |  |  |  |  |  |  |  |  |  | *89 93* |  |  |  |  | 278 278 |  |  |
